# Stabilizing Effect of Various Polyols on the Native and the Denatured States of Glucoamylase

**DOI:** 10.1155/2013/570859

**Published:** 2013-09-18

**Authors:** Mohammed Suleiman Zaroog, Habsah Abdul Kadir, Saad Tayyab

**Affiliations:** Biomolecular Research Group, Biochemistry Programme, Institute of Biological Sciences, Faculty of Science, University of Malaya, 50603 Kuala Lumpur, Malaysia

## Abstract

Different spectral probes were employed to study the stabilizing effect of various polyols, such as, ethylene glycol (EG), glycerol (GLY), glucose (GLC) and trehalose (TRE) on the native (N), the acid-denatured (AD) and the thermal-denatured (TD) states of *Aspergillus niger* glucoamylase (GA). Polyols induced both secondary and tertiary structural changes in the AD state of enzyme as reflected from altered circular dichroism (CD), tryptophan (Trp), and 1-anilinonaphthalene-8-sulfonic acid (ANS) fluorescence characteristics. Thermodynamic analysis of the thermal denaturation curve of native GA suggested significant increase in enzyme stability in the presence of GLC, TRE, and GLY (in decreasing order) while EG destabilized it. Furthermore, CD and fluorescence characteristics of the TD state at 71°C in the presence of polyols showed greater effectiveness of both GLC and TRE in inducing native-like secondary and tertiary structures compared to GLY and EG.

## 1. Introduction

Maintenance of native conformation (folded state) is an important determinant for protein stability. However, native conformation is marginally stable over to its denatured counterpart, and this has been attributed to acquiring a unique three-dimensional structure by a protein in solution [1]. Therefore, protein stability subject has become an important issue for scientists to search for possible ways to increasing the stability of proteins in aqueous solution as they are generally used in industrial, medical, and pharmaceutical sectors. One of the popular strategies used to increase protein stability is the employment of cosolvents (osmolytes), which are small organic molecules such as sugars, polyols, and neutral amino acids, favoring the folded ensemble. Recently, various studies have shown increase in thermal stability, gelation, foaming, and emulsion-stabilizing performance of globular proteins in presence of these osmolytes [2–4].

Different molecular characteristics of the cosolvents such as size, structure, and their interactions with other solvent molecules have been suggested to translate their effectiveness to alter the properties of globular proteins in aqueous solution. Furthermore, the type and the amount of the cosolvent play a critical role in modulating protein functionality, that is, enhancing conformational stability of the protein against certain environmental stresses such as exposure to extreme (high and low) temperature, high pressure treatment, or dehydration as well as obtaining an appropriate conformational state of the protein [5–7]. Although employment of cosolvents such as salts, amino acids, and polyols is a routinely used strategy to enhancing protein stability, the mechanism by which these cosolvents stabilize native protein conformation is still debatable. Numerous models have been proposed to explain the molecular basis of polyol-induced protein stabilization such as preferential interaction and surface tension [8], excluded volume effect [9], transfer free energy of protein's chemical groups [10], and Wyman linkage function [11]. Preferential interaction theory emphasizes the role of preferential interactions between the protein surface and the cosolvent-solvent molecules [5–7] through either the steric exclusion or differential interactions [5, 12] in the polyol-induced structural stabilization of globular proteins in aqueous solutions. Most of the studies on polyol-induced protein stabilization have resided on the role of polyols in terms of their chemical nature and concentration requirement in inducing protein stability. Importance of protein's intrinsic factors such as size, charge, and chemical composition has rarely been attended in polyol-induced stabilization of proteins. In view of the above, there is a need to extend polyol-induced stabilization study on other proteins in order to generalize the stabilization mechanism.

Glucoamylase (GA) (EC 3.2.1.3), an industrial enzyme is employed for the commercial production of glucose from starch, which is used in the preparation of fructose syrup and ethanol [13, 14]. GAs from *Aspergillus niger* and *Rhizopus oryzae* have received industrial preferences due to their higher stability and activity [15]. GA from *Aspergillus niger* is a glycoprotein with a molecular mass of 97 KD and is composed of 616 amino acid residues, arranged in a linear polypeptide chain. Three-dimensional structure of *Aspergillus niger* GA shows the presence of two domains, an N-terminal catalytic domain (1–440) and a C-terminal starch binding domain (SBD) (509–616), connected by a glycosylated linker region [16, 17]. The two isoforms of GA can be differentiated on the basis of SBD region, which is absent in GA II [17]. Presence of 12 *α*-helical segments, forming *α*/*α* barrel and six 3_10_-helices, characterizes the catalytic domain whereas the SBD is rich in one parallel and six antiparallel pairs of *β*-strands forming an open-sided *β*-barrel [18, 19]. The effect of different denaturants such as temperature, guanidine hydrochloride (GdnHCl), and urea on the conformation and activity of GA has been studied [20–22]. In a previous study, we have characterized the AD state of *Aspergillus niger* GA at pH 1.0 as a molten globule-like state [23]. Transformation of the molten globule-like state into the partially folded state will add towards our understanding about folding of a particular protein. Polyols are well-known protein stabilizers [5–7, 12]. However, the effect of polyols on the structure and stability of the N state and the AD state of GA has not been studied so far to the best of our knowledge. In this report, we present our results on the effect of four cosolvents (polyols) including two sugars (glucose (GLC) and trehalose (TRE)) and two alcohols (glycerol (GLY) and ethylene glycol (EG)) on the conformation and stability of the N state and the AD state at pH 1.0 using different probes such as, far-UV CD spectral signal, ANS fluorescence, and Trp fluorescence. Furthermore, thermal denaturation data of the N state of GA in the absence and the presence of these polyols are also included.

## 2. Materials and Methods

### 2.1. Materials

Glucoamylase from *Aspergillus niger* (Lot 1390149), 1-anilinonaphthalene-8-sulfonic acid (Lot 104K2510), trehalose (Lot 011M7000V), glucose (Lot 080M0175V), glycerol (Lot SHBB4673V), and ethylene glycol (Lot STBB0339H9) were procured from Sigma-Aldrich Inc., USA. Analytically pure samples of other chemicals were used. Acid-denatured (AD) state of GA was prepared following the procedure described earlier [23].

### 2.2. Analytical Procedures

Spectrophotometric method, using a molar extinction coefficient of 1.37 × 10^5^ M^−1 ^cm^−1^ at 280 nm, and the method of Lowry et al. [24] were employed to determine GA concentration.

ANS concentration was determined spectrophotometrically, using a molar extinction coefficient of 5 × 10^3^ M^−1 ^cm^−1^ at 350 nm [25].

### 2.3. Far-UV CD Spectroscopy

Jasco spectropolarimeter, model J-815, fitted with a thermostatically-controlled cell holder and linked to a water bath, was used for CD measurements under constant nitrogen flow. The solution of (+)-10-camphorsulfonic acid was used to calibrate the instrument, and a scan speed of 50 nm/min with a response time of 1 s was fixed for CD measurements at 25°C. The spectral measurements were made in the far-UV region (200–250 nm) using a protein concentration of 1.4 *μ*M, taken in a 1 mm path length cuvette. Each spectrum was recorded in triplicate, and the average of three scans was corrected with appropriate blanks. The transformation of CD data into mean residue ellipticity, MRE values in deg·cm^2^·dmol^−1^, was made following the procedure described elsewhere [26].

### 2.4. Fluorescence Spectroscopy

Fluorescence spectra were recorded on a Jasco spectrofluorometer, model FP-6500, attached to a data recorder and supplied with a thermostatically-controlled cell holder at 25°C or 71°C. The protein solution (0.12 *μ*M), taken in a 1 cm path length cuvette, was excited at 295 nm, and the emission spectra were recorded in the wavelength range of 310–400 nm, using a slit width of 10 nm for both excitation and emission wavelengths.

In ANS fluorescence experiments, fluorescence spectra were recorded in the wavelength range of 400–600 nm while the excitation wavelength was set at 380 nm, using a protein concentration of 0.26 *μ*M. The molar ratio between the ANS and the protein was fixed at 70 : 1.

### 2.5. Thermal Denaturation

The effect of temperature on native GA both in the absence and the presence of different polyols was studied by measuring mean residue ellipticity at 222 nm (MRE_222 nm_) in the temperature range of 20–100°C. A scan rate of 1°C min^−1^ was used throughout the temperature range, while other experimental conditions were maintained similar to those described above. Thermal denaturation curves were analyzed using two-state model [23].

### 2.6. Structural Changes in GA in the Presence of Various Polyols

The structural changes in the native and the acid-denatured GAs in the presence of various polyols were studied following the method described by Devaraneni et al. [27]. To 0.5 mL stock protein solution dissolved in water (14 *μ*M for CD and 1.2 *μ*M for fluorescence measurements), 4.5 mL of the buffer (10 mM glycine-HCl buffer, pH 1.0, or 10 mM sodium phosphate buffer, pH 7.0) containing the desired polyol concentration was added. The contents in each tube were mixed gently, and the mixture was incubated for 12 h at 25°C before CD/fluorescence measurements. Blank solutions were prepared in the same way except that the protein solution was replaced with the suitable buffer. Far-UV CD spectral signal was employed to monitor the secondary structural changes in the presence of various polyols, whereas the change in the tertiary structure was studied by Trp fluorescence and ANS fluorescence measurements. Furthermore, MRE_222 nm_ measurements were also used to study the stabilizing effect of various polyols on the native GA.

## 3. Results and Discussion

### 3.1. Polyol-Induced Structural Changes in the Native and the Acid-Denatured GAs

The effects of various cosolvents (polyols) including a monosaccharide (GLC), a disaccharide (TRE), a dihydric alcohol (EG), and a trihydric alcohol (GLY) on the conformation of the N and the AD states of glucoamylase were studied using different probes such as far-UV CD, Trp fluorescence, and ANS fluorescence.

#### 3.1.1. Far-UV CD Spectra

**Figure 1:**
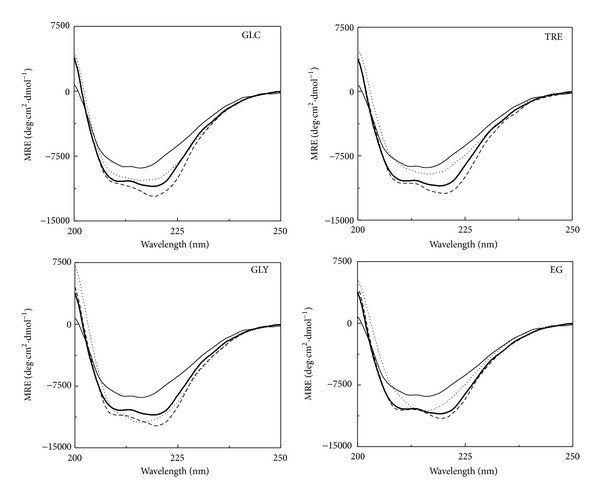
Effect of various polyols, such as, glucose (GLC), trehalose (TRE), glycerol (GLY), and ethylene glycol (EG), on the far-UV CD spectra of the native and the acid-denatured GAs. Different line symbols represent native GA (thick curve), acid-denatured GA (thin curve), native GA + polyol (dashed curve), and acid-denatured GA + polyol (dotted curve). The spectra were recorded at 25°C using a protein concentration of 1.4 *μ*M. Different polyol concentrations used were 2.6 M GLC, 1.3 M TRE, 8.0 M GLY, and 8.0 M EG.

Polyol-induced secondary structural changes in the N and the AD states of GA were studied using far-UV CD spectroscopy.  Figure 1 shows far-UV CD spectra of the N and the AD states of GA, obtained at 25°C both in the absence and the presence of various polyols (GLC, TRE, GLY, and EG). As can be seen from Figure 1, far-UV CD spectrum of the N state was characterized by the presence of two negative signals around 210 and 219 nm, characteristic of the *α*-helical structure of the protein [28]. On the other hand, far-UV CD spectrum of the AD state showed a significant decrease in the MRE values along with a shift in the wavelength of the negative signals, which occurred at 212 and 216 nm. Both these changes indicated a different conformation (with less *α*-helical structure) of the AD state of GA compared to the N state. In a previous report, we have characterized the AD state of GA at pH 1.0 as the molten globule-like state [23]. Addition of polyols to the N and the AD states of GA produced structural changes in both states as reflected from the increase in the MRE values, being more pronounced in the AD state than the N state (Figure 1). Quantitative analysis of these results in terms of MRE_222 nm_ values of the two states of GA obtained in the absence and the presence of various polyols along with the percentage increase in the MRE_222 nm_ value (ΔMRE_222 nm_) in the presence of polyols is given in Table 1. Whereas the presence of GLY produced a maximum increase in the MRE_222 nm_ of ~47% in the AD state, only ~14% increase in the MRE_222 nm_ was observed in the N state. These results were similar to various reports on other proteins where the effect of different polyols on the N state was found relatively lesser than that observed with the AD state [29, 30]. A comparison of polyol-induced conformational changes in the N and the AD states of GA, based on ΔMRE_222 nm_, showed greater effectiveness of GLY in both states. On the other hand, EG and TRE were found least effective in altering the secondary structures in the N state and the AD state, respectively. Quantitatively, various polyols showed the order of effectiveness in increasing MRE_222nm_ value in the AD state as GLY > GLC > EG > TRE (Table 1).

In addition to the increase in MRE_222 nm_ value of both states of GA in the presence of different polyols, change in the shape of the CD spectrum was also noticed. Although the characteristic shape of the CD spectrum showing *α*-helical structure was retained in the CD spectra of the N state in the presence of polyols, shape of the CD spectrum of the AD state was transformed into a CD spectrum showing characteristics of *β*-structure in the presence of all polyols except GLC (Figure 1). Since the catalytic domain of GA is rich in the *α*-helical segments while *β*-structure is more populated in the SBD [18, 19], these polyols seem to induce structural changes in both domains to a different extent in the AD state. On the other hand, polyols might have produced structural changes restricted to the catalytic domain of GA in its N state.

#### 3.1.2. Trp Fluorescence Spectra

**Figure 2:**
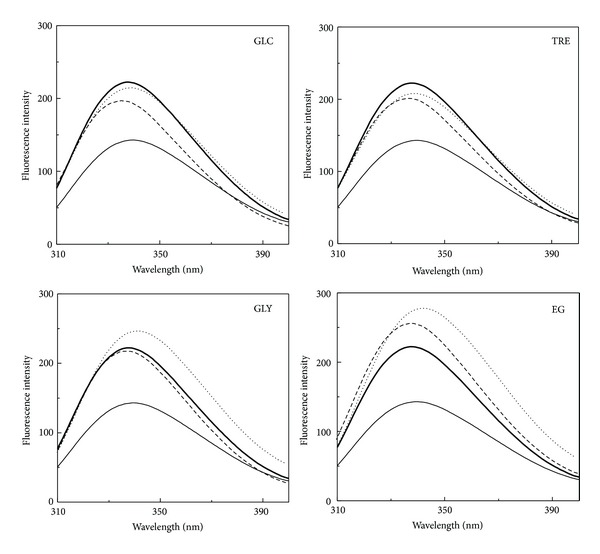
Effect of various polyols, such as, glucose (GLC), trehalose (TRE), glycerol (GLY), and ethylene glycol (EG), on the tryptophan fluorescence spectra of the native and the acid-denatured GAs. Different line symbols represent native GA (thick curve), acid-denatured GA (thin curve), native GA + polyol (dashed curve), and acid-denatured GA + polyol (dotted curve). The spectra were recorded at 25°C using a protein concentration of 0.12 *μ*M. Different polyol concentrations used were 2.6 M GLC, 1.3 M TRE, 8.0 M GLY, and 8.0 M EG.

In order to study tertiary structural changes in the N and the AD states of GA in presence of polyols, Trp fluorescence was employed to monitor the microenvironment around Trp residues. The fluorescence spectra of the N and the AD states of GA both in the absence and the presence of different polyols are shown in Figure 2. As evident from the figure, fluorescence spectrum of the N state of GA was characterized by the presence of an emission maximum (*E*_*m*_) at 340 nm, when excited at 295 nm. About 36% decrease in the fluorescence intensity (FI) along with 2 nm red shift in the *E*_*m*_ was noticed in the fluorescence spectrum of the AD state (Table 2). These results were in agreement with our previous results on the AD state of GA [23]. A marked increase (46–94%) in the FI at 342 nm (FI_342 nm_) of the AD state of GA was observed in the presence of polyols, following the order EG > GLY > GLC > TRE (Table 2), which was suggestive of the transfer of Trp residues from a polar environment to a nonpolar environment in the presence of these polyols. In addition to an increase in the FI_342 nm_, a slight red shift (1-2 nm) in the *E*_*m*_ was also observed in the presence of EG and GLY whereas GLC and TRE showed a small blue shift (1-2 nm) in the *E*_*m*_ (Table 2). These results suggested partial refolding of the AD state of GA in the presence of these polyols, marked by the burial of Trp residues in nonpolar interior of the protein. In view of the distribution of Trp residues in the catalytic domain (13 Trp) and SBD (4 Trp) [16], change in the FI_342 nm_ of the protein mainly reflected the structural changes in the catalytic domain. Furthermore, these results were supported by the far-UV CD spectral results, showing increased *α*-helical structure in the AD state of GA in the presence of polyols (Table 1).

On the other hand, presence of GLY, GLC, or TRE in the incubation mixture containing native GA produced a small quenching (2–12%) in the fluorescence intensity, suggesting partial exposure of Trp residues to the polar environment. Contrary to it, EG showed similar behavior with the N state as that shown with the AD state by producing an increase (15%) in the FI_340 nm_ (Table 2). The increase in the fluorescence intensity of the N state in the presence of EG against the decrease shown in the presence of other polyols indicated different conformational structures acquired by the catalytic domain in the presence of these polyols.

#### 3.1.3. ANS Fluorescence Spectra

**Figure 3:**
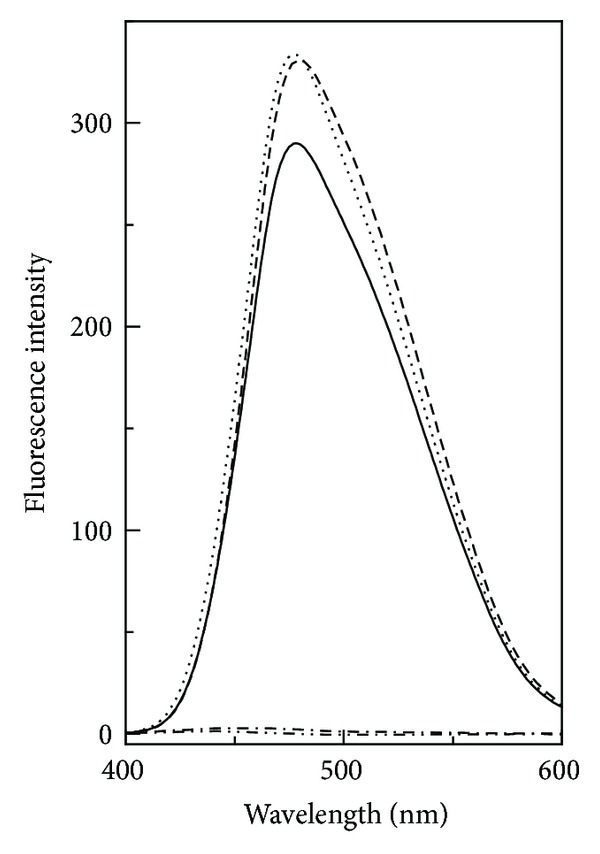
ANS fluorescence spectra of the acid-denatured GA in the absence (thick curve) and the presence of 2.6 M GLC (dashed curve), 1.3 M TRE (dotted curve), 8 M GLY (dashed with one dot curve), and 8 M EG (dashed with two dots curve). The spectra were recorded at 25°C, using a protein concentration of 0.26 *μ*M and ANS : protein molar ratio as 70 : 1.

Binding of a hydrophobic dye, ANS, to the AD state of GA in the absence and the presence of various polyols was studied to get insight about the tertiary structural changes in the AD state induced by these polyols and the results are shown in Figure 3. Native GA produced a weak ANS fluorescence spectrum with an *E*_*m*_ at 470 nm (figure omitted for brevity), indicating burial of the hydrophobic regions in the protein interior in the N state [31, 32]. The AD state showed a marked increase in the ANS fluorescence intensity along with 8 nm red shift in the *E*_*m*_ (Figure 3), suggesting exposure of the protein's hydrophobic segments to the solvent at pH 1.0. These results agreed well with those reported earlier for acid-denatured proteins [27, 33]. Presence of 2.6 M GLC or 1.3 M TRE in the incubation mixture led to a further increase in the FI at 478 nm by 14% and 15%, respectively, accompanied by 2 nm red shift in the *E*_*m*_ for GLC and 1 nm blue shift in the *E*_*m*_ for TRE, suggesting the availability of more hydrophobic clusters to the solvent. Interestingly, 8.0 M GLY or 8.0 M EG completely quenched the ANS fluorescence, similar to that found with the N-state, indicating burial of the hydrophobic segments, which were solvent-exposed in the AD state. As a large number of hydrophobic residues are predominantly distributed in the SBD region [16], burial or exposure of these residues mainly reflected structural alteration in the SBD. In view of the burial of the hydrophobic segments upon addition of GLY or EG and more exposure in the presence of GLC or TRE, it appears that different conformational makeup was acquired in the SBD in the presence of these polyols. These results agreed well to our far-UV CD spectral results where both GLY and EG induced more *β*-structural features (Figure 1).

### 3.2. Polyol-Induced Thermal Stabilization of GA

#### 3.2.1. Thermal Transition

**Figure 4:**
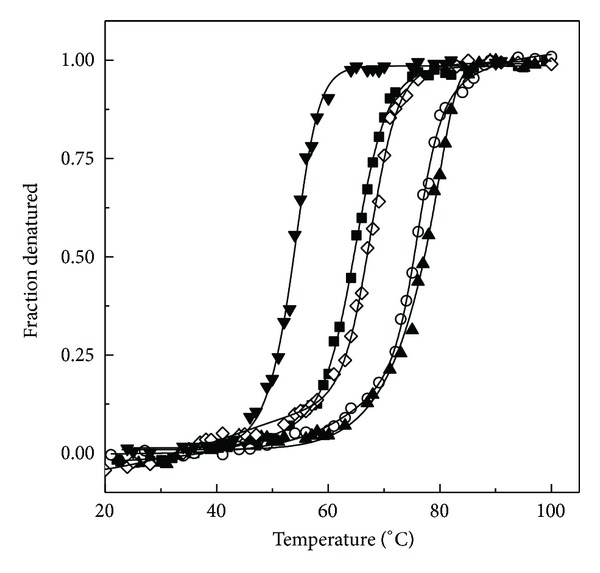
Normalized thermal transition curves of the native GA in the absence (■) and the presence of 2.6 M GLC (▲), 1.3 M TRE (○), 8 M GLY (*◊*), and 8 M EG (*▼*) as monitored by MRE_222 nm_ measurements, using a protein concentration of 1.4 *μ*M.

Figure 4 shows normalized transition curves (*F*_*D*_ plots) of thermal denaturation of GA in the absence and the presence of various polyols as studied by MRE_222 nm_ measurements. As evident from the figure, MRE_222 nm_ of GA remained unchanged within 20–47°C, decreased markedly between 53°C and 77°C, and became constant thereafter up to 100°C. Thermal denaturation of GA showed loss of the secondary structure with increasing temperature in a cooperative manner. Several proteins have shown thermal transition as a cooperative process [33–35]. Thermal transition curves of GA obtained in the presence of GLC, TRE, or GLY were found shifted toward the higher temperature range, suggesting increased thermal stability of GA in the presence of these polyols. Contrary to it, presence of EG in the incubation mixture shifted the thermal transition curve toward the lower temperature range, indicating destabilization of GA. Previous studies have also shown stabilization of many proteins in the presence of GLC, TRE, and GLY [36–38] and destabilization in the presence of EG [27, 39].

Thermodynamic analysis of the transition curves was made as described in the Materials and Methods, and the values of *T*_*m*_, Δ*H*_*vH*_, and Δ*G* (25°C), thus obtained, are presented in Table 3. The midpoint temperature, *T*_*m*_ (64.3°C) obtained for GA, was found similar to an earlier report [23]. As can be seen from the table, presence of GLC, TRE, or GLY in the incubation mixture increased the stability of GA whereas EG destabilized it. Glucose was found as the strongest cosolvent in increasing thermal stability of GA among the polyols studied, as it increased the *T*_*m*_ value by 13°C up to 77°C along with 80% increase in the Δ*G* (25°C). In general, a comparison of different polyols based on thermodynamic parameters, shown in Table 3, suggested the effectiveness order as GLC > TRE > GLY for GA stabilization. This order was found similar to that reported earlier for yeast hexokinase A [27] and *α*-amylase [33].

#### 3.2.2. Effect of Polyols on the Thermal-Denatured GA at 71°C

**Figure 5:**
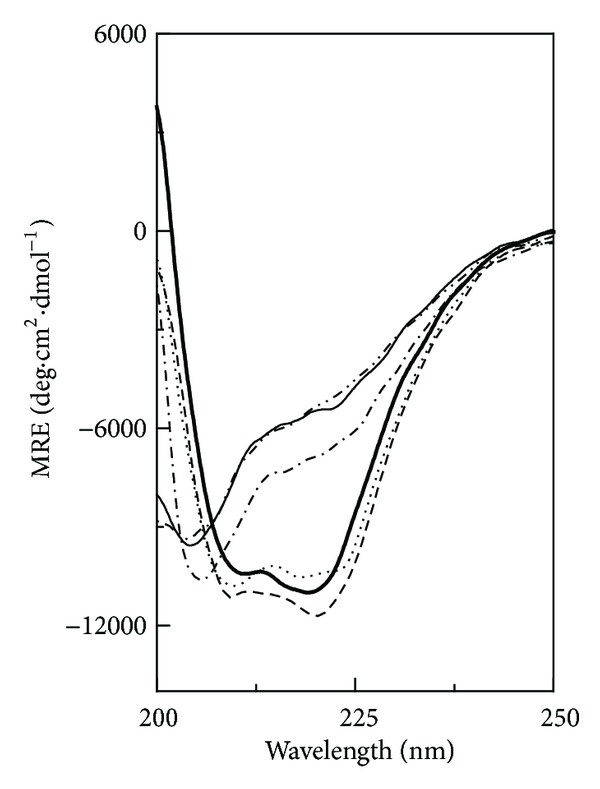
Far-UV CD spectra of the native (thick curve) and the thermal-denatured GAs in the absence (thin curve) and the presence of 2.6 M GLC (dashed curve), 1.3 M TRE (dotted curve), 8 M GLY (dashed with one dot curve), and 8 M EG (dashed with two dots curve). The spectra of the thermal-denatured GA were recorded after equilibrating the sample at 71°C for 6 min, using a protein concentration of 1.4 *μ*M.

*Far-UV CD Spectra.* In order to verify the thermal stabilizing effect of these polyols, far-UV CD spectra of the thermal-denatured (TD) GA at 71°C were obtained in the absence and the presence of polyols (Figure 5). Far-UV CD spectrum of the native GA (pH 7.0, 25°C) has also been included in Figure 5 for comparison. As can be seen from the figure, far-UV CD spectrum of the TD state of GA showed ~48% loss in the MRE_222 nm_ value compared to the native GA (Tables 1 and 4), along with a shift in the CD spectral signals, suggesting denaturation of GA at high temperature as observed with other proteins [30, 36, 40]. Interestingly, addition of GLC or TRE to the TD state of GA markedly increased the MRE_222 nm_ value by ~110% and ~91%, respectively (Table 4), showing significant reversal in the CD spectral characteristics close to the native GA. However, slight change in the position of the minima was observed with TRE. On the other hand, no increase in the MRE_222 nm_ was observed in the presence of EG, rather it showed a ~6% decrease in the MRE_222 nm_ (Figure 5), suggesting no stabilizing effect of EG on the secondary structural characteristics of thermal-denatured GA. GLY was able to induce ~22% regain in the MRE_222 nm_ value. Thus, both GLC and TRE were able to induce native-like secondary structures in the thermal-denatured GA.

**Figure 6:**
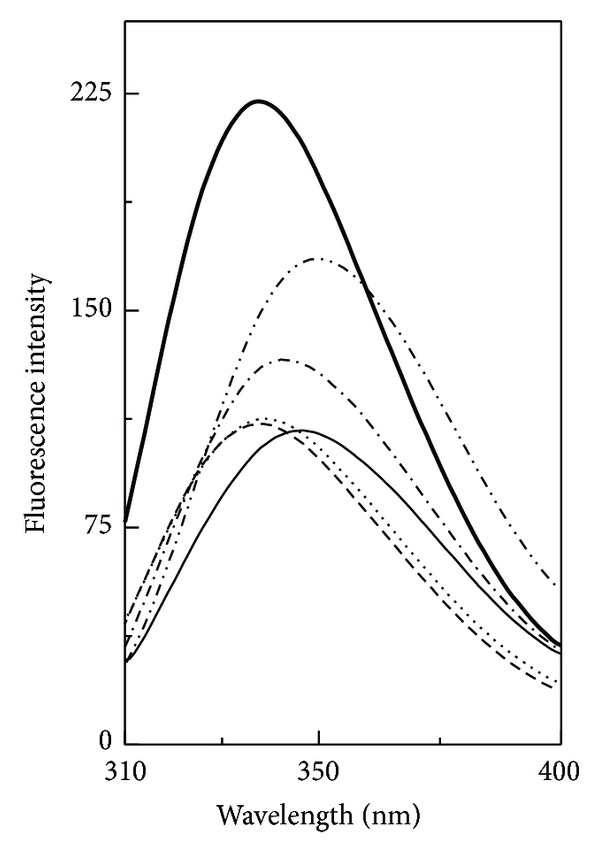
Tryptophan fluorescence spectra of the native (thick curve) and the thermal-denatured GAs in the absence (thin curve) and the presence of 2.6 M GLC (dashed curve), 1.3 M TRE (dotted curve), 8 M GLY (dashed with one dot curve) and 8 M EG (dashed with two dots curve). The spectra of thermal-denatured GA were recorded after equilibrating the sample at 71°C for 6 min, using a protein concentration of 0.12 *μ*M.

*Tryptophan Fluorescence Spectra.* Figure 6 shows Trp fluorescence spectra of the thermal-denatured GA at 71°C in the absence and the presence of polyols. Tryptophan fluorescence spectrum of the native GA is also included in Figure 6. Thermal-denatured GA showed a significant decrease (54%) in the FI_340 nm_, accompanied by 9 nm red shift in the *E*_*m*_, compared to the native GA (Tables 2 and 4). Both decrease in the FI_340 nm_ and significant red shift in the *E*_*m*_ of the TD state of GA were suggestive of the exposure of the Trp residues to the polar solvent [41], indicating protein denaturation. Except EG, other polyols (GLC, TRE, and GLY) produced a significant blue shift (5–9 nm) and increase in the FI_340 nm_. Both these changes in the fluorescence characteristics of the TD GA suggested significant refolding in the enzyme, characterized by the burial of the Trp residues in the nonpolar environment. Both GLC and TRE were found more effective in inducing native-like tertiary structure as reflected from the retrieval of the *E*_*m*_, similar to the *E*_*m*_ of the native GA. These results were similar to the far-UV CD spectral results (Figure 5), where both GLC and TRE were found to induce secondary structure, similar to that present in the native GA. Presence of a polar/charged group in the vicinity of the Trp residues might account for the lesser increase in the FI_340 nm_ in the presence of GLC and TRE [42]. On the other hand, addition of EG to the incubation mixture produced only 3 nm red shift in the *E*_*m*_ along with a marked increase (45%) in the FI_340 nm_, indicating a different tertiary structural make-up, compared to the one obtained with other polyols. The far-UV CD spectral signal of the thermal-denatured GA in the presence of EG also showed no significant change in the CD spectral characteristics (Figure 5).

## 4. Conclusions

Taken together, all polyols appeared to induce the native-like structure (to a greater extent) in the acid-denatured GA. This was evident from the higher MRE_222 nm_ and FI_342 nm_ values of the acid-denatured GA in the presence of polyols compared to those obtained in their absence (Tables 1 and 2). Polyols were also found to stabilize the native state against thermal denaturation. On the other hand, EG produced the opposite effect.

## Figures and Tables

**Table 1:** Effect of various polyols on the MRE_222 nm_ of different states of GA.

Polyols	N state (pH 7.0, 25°C)		AD state (pH 1.0, 25°C)	
	MRE_222 nm_	*ΔMRE_222 nm_ (%)	MRE_222 nm_	ΔMRE_222 nm_ (%)
—	−10500	—	−7400	—
2.6 M GLC	−11683	11.3	−9637	30.2
1.3 M TRE	−11664	11.1	−8570	15.8
8.0 M GLY	−11936	13.7	−10858	46.7
8.0 M EG	−11018	4.9	−8948	20.9

*ΔMRE_222 nm_ represents percentage change in the MRE_222 nm_ in the presence of polyols.

**Table 2:** Effect of various polyols on the fluorescence spectral characteristics of different states of GA.

Polyols	N state (pH 7.0, 25°C)			AD state (pH 1.0, 25°C)		
	^ 1^ *E*_*m*_ (nm)	^ 2^FI_340 nm_	^ 3^ΔFI (%)	*E*_*m*_ (nm)	FI_342 nm_	ΔFI (%)
—	340	222	—	342	143	—
2.6 M GLC	338	195	−12.2	341	214	49.7
1.3 M TRE	338	201	−9.5	340	208	45.5
8.0 M GLY	339	218	−1.8	343	247	72.7
8.0 M EG	339	256	+15.3	344	277	93.7

^1^ *E*_*m*_: Emission maxima.

^2^FI: Fluorescence intensity at 340/342 nm.

^3^ΔFI: Percentage change in the fluorescence intensity in the presence of polyols.

**Table 3:** Thermodynamic parameters for thermal denaturation of glucoamylase as monitored by CD spectroscopy.

Glucoamylase	*T*_*m*_ (°C)	Δ*H* (KJ mol^−1^)	Δ*G* (25°C) (KJ mol^−1^)	ΔΔG^a^ (%)
Native (N)	64.3	264.30	30.79	—
N + 2.6 M GLC	77.2	365.56	55.52	80.3
N + 1.3 M TRE	75.3	326.77	48.12	56.3
N + 8.0 M GLY	66.9	283.72	36.74	19.3
N + 8.0 M EG	53.2	307.02	26.48	−14.0

^a^ΔΔ*G* represents percentage change in the Δ*G* value in the presence of polyols. Negative sign shows the decrease.

**Table 4:** Effect of various polyols on the CD and fluorescence spectral characteristics of the thermal-denatured GA at 71°C.

Polyols	CD		Fluorescence		
	MRE_222 nm_	^ 1^ΔMRE_222 nm_ (%)	^ 2^ *E*_*m*_ (nm)	^ 3^FI_340 nm_	^ 4^ΔFI (%)
—	−5420	—	349	103	—
2.6 M GLC	−11404	110.4	340	111	7.8
1.3 M TRE	−10360	91.1	341	113	9.7
8.0 M GLY	−6624	22.2	344	130	26.2
8.0 M EG	−5104	−5.8	352	149	44.7

^1^ΔMRE_222 nm_ represents percentage change in the MRE_222 nm_ in the presence of polyols. Negative sign shows the decrease.

^2^ *E*_*m*_ = Emission maxima.

^3^FI_340 nm_ = Fluorescence intensity at 340 nm.

^4^ΔFI = Percentage change in the fluorescence intensity in the presence of polyols.

## Abbreviations

ANS: 1-Anilinonaphthalene-8-sulfonic acid
CD: Circular dichroism
EG: Ethylene glycol
GA: Glucoamylase
GLC: Glucose
GLY: Glycerol
MRE: Mean residue ellipticity
SBD: Starch binding domain
TRE: Trehalose
Trp: Tryptophan.
